# Exploring the flavor life cycle of beers with varying alcohol content

**DOI:** 10.1002/fsn3.472

**Published:** 2017-04-20

**Authors:** Benjamin Missbach, Dorota Majchrzak, Raphael Sulzner, Brian Wansink, Martin Reichel, Juergen Koenig

**Affiliations:** ^1^ Department of Nutritional Sciences University of Vienna Vienna Austria; ^2^ Charles H. Dyson School of Applied Economics and Management Cornell University Ithaca New York

**Keywords:** Alcohol‐free beer, alcohol‐reduced beer, flavor attributes, regular beer, temporal dominance of sensations, worty off‐flavor

## Abstract

Decreasing alcohol in beer and increasing the pleasure of lower alcohol beers is a potential way to limit total alcohol consumption. Consumers’ willingness to drink alcohol‐reduced beers is mainly limited by unfavorable flavor characteristics that arise during consumption. To investigate the temporal flavor dominance during consumption, we analyzed the five most dominant beer flavors from nine different beers among three types of beer with varying alcohol content to assess the Flavor Life Cycle. Results show that beers with different alcohol content displayed similar flavor dominance (e.g., bitterness) and displayed differences in worty‐off flavor, malty flavor, and astringency. In alcohol‐free beers, worty‐off flavor was most pronounced in dominating between 5 and 30 s and malty flavor increased after swallowing. For bitterness and astringency, higher alcohol content resulted in higher flavor dominance, especially prior to swallowing (≤40 sec). Based on these findings, we provide some brief advice to minimize unfavorable flavor experience during consumption of beer with lower alcohol. For now, consumers who want to enjoy beers with lower alcohol should consider flavor changes and focus on the favored and defocus on the less favored flavors.

## 1 INTRODUCTION

Beer is one of the most popular beverages in Europe (Shield, Rylett, & Rehm, 2016) and in recent years, sales numbers for beers with reduced alcohol have increased (Brányik, Silva, Baszczyňski, Lehnert, & e Silva, 2012). Despite this trend, some constraints perpetuate their consumption, such as problematic sensory characteristics that emerge during beer consumption (Flavor Life Cycle). To identify dominant flavors in the Flavor Life Cycle and to overcome potential sensory problems will help consumers make more responsible drinking choices and can help on a population‐level, likewise. For this, we will explore the most dominant sensory attributes (beer flavors) during consumption of different types of beers, using a dynamic sensory method.

Regular beers vary between 3% and 6% alcohol by volume. Reducing the alcohol content in beers result in products that are labeled as alcohol‐reduced beers, or alcohol‐free beers (≤0.5% alcohol by volume). Previous research reported increasing worldwide market share for alcohol‐reduced and alcohol‐free beers (Brányik et al., 2012). For this, many major breweries have expanded their portfolio for beers with lower alcohol content (Seekingalpha, 2016). Beer shows a highly complex sensory profile (Meilgaard, Dalgliesh, & Clapperton, 1979). Traditionally, beer is brewed from barley, malt, hops, and water in a multi‐step brewing and fermentation process. Alcohol‐reduced and alcohol‐free beer production warrants specific technological requirements (Liguori et al., 2015; Sohrabvandi, Mousavi, Razavi, Mortazavian, & Rezaei, 2010), while the main goal is to achieve a sensory profile matrix alike conventional beer products (Burberg & Zarnkow, 2009). Mapping the sensory profile of regular beer demonstrated that several different flavor attributes containing >800 active compounds compose the complex beer flavor during consumption (Parker, 2012). Astringency, fruity, bitterness, and malty are considered to be important for consumers’ acceptance of beer (Porretta & Donadini, 2008). During fermentation processes, wort‐fermented volatile compounds contribute to this complex beer flavor (Liguori et al., 2015). Research showed that the anticipated sensory profile contributes to consumer expectations, eventually determining beer acceptance during and after consumption (Catarino, Ferreira, & Mendes, 2009). It is noted by Sester, Dacremont, Deroy, and Valentin (2013) that *‘‘consumers expect to find flavors, such as bitterness, texture, such as sparkles or physiological quality, such as being thirst‐quenching”* and they also speculate *“that a beer could be rejected if these expectations are not confirmed’’* (Sester et al., 2013, P. 480). Previous research also showed that, compared to conventional beer, alcohol‐reduced and alcohol‐free beers display several problems that emerge during beer production: (1) freezing problematic; (2) improper foaming issues; (3) increased microbial contamination; (4) immature flavor profile and mouth feeling; (5) off‐flavors, that are associated with the reduction or elimination of alcohol content (Sohrabvandi et al., 2010). Especially the flavor profile, mouth feeling and off‐flavor are considered to shape consumer acceptance most prominently, but these problematic issues have yet to be solved. To do so, it is an important first step to describe the most relevant flavor attributes during consumption (Flavor Life Cycle) of beers with reduced alcohol content.

Consumers’ ability to discriminate between beverages with varying alcohol‐content has been reported in the literature, both between alcohol‐free and regular beer (Lachenmeier, Kanteres, & Rehm, 2014) and between alcohol‐reduced and regular beers (Segal & Stockwell, 2009). As noted by Lachenmeier et al. (2014), consumers are able to discriminate between alcohol‐free and regular beers, however they could not distinguish between beer displaying higher alcohol by volume. Besides the ability to discriminate between beers with varying alcohol, identifying the main flavor attributes that deviate from the expected beer flavor is important, because this potentially limits consumer acceptance. In this study, trained panelists assess the most dominant flavor attributes across beers with varying alcohol content. To date, different brands have added alcohol‐reduced and alcohol‐free beers to their portfolio that will allow us to compare both within‐ and between brand differences in temporal flavor dominance during the Flavor Life Cycle. For this, we use a dynamic sensory method (Temporal Dominance of Sensations: TDS) to analyze the temporal differences in flavor dominance during consumption.

This study will help consumers, breweries, and Public Health stakeholders likewise. Identifying critical flavor attributes that drive or diminish consumer acceptance during consumption might help improve product development. With this, breweries can adapt their technological processes, driven by bottom‐up consumer profiling. For consumers, this feeds back into improved and more similar products and increases consumer acceptance for alcohol‐reduced beers. In addition, this research informs consumers of what to expect during consumption of beers with different alcohol content and helps them maximize their consumption experience. From a Public Health perspective, reducing the alcohol content in various beverages has been proposed as a potential strategy to decrease the burden of alcohol‐associated risk and disease (Rehm, Lachenmeier, Llopis, Imtiaz, & Anderson, 2016). Modifying beers by reducing the alcohol content without taking away the pleasure of consumption is an important opportunity to move forward in this area of Public Health (Shield et al., 2016).

During consumption, no previous study investigated the most dominant flavor attributes of beers with varying alcohol content within and between brands. Analyzing the temporal Flavor Life Cycle of the most important flavors for beer consumers (bitterness, astringency, fruity, malty, and worty) will allow us to draw conclusions for potential modifications of beers and maximize consumer satisfaction.

## 2 MATERIALS AND METHODS

We applied a 3 (beer brand) × 3 (alcohol content) between‐subjects design with 10 trained panelists (regular beer drinkers) to assess temporal change in flavor perception. To evaluate the temporal change in flavor dominance of the five most important attributes to consumers (worty, fruity, bitter, astringency, malty), we tested three different types of beer from three different brands by applying the Temporal Dominance of Sensations method (TDS). In Table 1, differences in ethanol content are displayed. Regular beers varied between 4.9% and 5.4% alcohol by volume and were all “Märzen” or lager beer with light to brown color. Alcohol‐reduced beers contained 3.0–3.5% alcohol by volume, while in alcohol‐free beers, ethanol content was <0.5% alcohol by volume. Both displaying similar coloring like regular beers.

**Table 1:** Alcohol volume of beer samples

	Beer Category	Vol % Ethanol
Brand A	Regular Beer	5.0
	Alcohol‐reduced Beer	3.5
	Alcohol‐free Beer	<0.5
Brand B	Regular Beer	5.4
	Alcohol‐reduced Beer	3.0
	Alcohol‐free Beer	<0.5
Brand C	Regular Beer	4.9
	Alcohol‐reduced Beer	3.3
	Alcohol‐free Beer	<0.5

All beers displayed the same production date and were served at the same temperature.

Sensory analysis was conducted in individual taste‐booths at the Sensory Laboratory of the Department of Nutritional Sciences (University of Vienna), designed and equipped according to the International Organization for Standardization (ISO, 1998). No incentives were given for participating in this study. Sensory analysis was performed for two days in fall 2015 and the study procedure adhered to the guidelines of the Declaration of Helsinki in its revised form 2008. We included panelists who had at least two years of experience in descriptive sensory languages and were regular beer drinkers with an age ranging from 24 to 35 years. Prior to testing, a four‐step training session for all panelists was mandatory:

1. Introduction to the TDS as a sensory method
2. Attribute familiarization using reference products (fruity: red fruit juice; bitter: caffeine or hop; astringency: black tea; malty: malt extract; worty: potato puree)
3. Training session in Software architecture and handling
4. Training in usage of the TDS to improve handling for further testing

To evaluate the dominant flavor attributes, we applied a TDS, which aims to record the evaluation of the dominant sensory perceptions of each product during consumption. Sensory evaluation was assisted by a computerized system, displaying all attributes to the panelist on a computer screen. During consumption, all panelists were free to select the most dominant attribute multiple times (100 s). Panelists were presented with three beer samples in a randomized order. All beers were served with the recommended serving temperature ranging from 7 to 11°C from a transparent shot glass (10 ml). Panelists were asked to push the “start” button once they sipped the first sample that activated the application software and started the recording time. They were then instructed to keep the sample in their mouth for the first 40 s prior to swallowing. All five attributes were displayed on the computer screen and were evaluated continuously. Panelists indicated the most salient and most dominant attribute each time they felt that the sensation has changed by clicking on the dedicated keyboard buttons (Pineau et al., 2009).

In this study, we investigated the four most dominant flavor attributes (bitter, astringency, malty, fruity), and one undesirable off‐flavor attribute (worty flavor). During the fermentation process with yeast, different ester compounds are produced (e.g., ethyl acetate and iso‐amyl acetate) responsible for fruity flavor like citrus, apple, banana, and blackcurrant (Meilgaard et al., 1979; Sohrabvandi et al., 2010). Astringency is a somatosensory perception described as throaty or constringent; it is tightly connected to the pH‐value present in beer, as higher acidity is associated with increased astringency. Malty flavor is a central attribute in beer flavor and depends on the type of malt used during beer processing (Taylor & Organ, 2009). As an undesired off‐flavor, the worty flavor is the most prominent flavor that is characterized by potato‐like unfermented wort. Several carbonyl compounds (e.g., 3‐methylbutanal, 2‐methylbutanal, and 3‐methylpropionaldehyde) with very low odor threshold values contribute to the worty off‐flavor (Perpete & Collin, 1999; Perpète & Collin, 2000; Perpete, Collin, & Collin, 2003). Worty off‐flavor arise when aldehydes – that are produced during wort boiling – are not reabsorbed by yeast cells and reduced to alcohol during dealcoholization (Sohrabvandi et al., 2010).

By using the TDS method, panelists were instructed to discern between the most dominant sensations of each product or product group (Pineau & Schlich, 2015). The TDS curves display differences between the tested samples over the time of consumption. To calculate the dominance rates across all panelists and sessions for each of the five flavor attributes, we divided the number of citations for each attribute by the total number of evaluations across all panelists. Increased dominance frequencies indicate higher agreement among panelists for each individual attribute. Data smoothing procedure was used to produce time‐dominance curves that include chance level and significance level lines. Chance level lines represent the dominance frequencies an attribute can obtain by chance. The chance line represents the statistical chance for one particular attribute being perceived as dominant. Displaying the chance level is necessary to calculate the significance level:

$$P_{0}=1/\text{number of attributes}$$

In this study, we presented five different attributes resulting in a calculated chance level of 20%. Significance level lines represent the lowest significant proportion value with α* < *0.05 at any given point in time. This line indicates the minimum value that must be reached for any attribute dominance to be considered as significantly higher than the chance level. The calculation is based on the number of replication sessions for each panelist per beer sample,

$$P_{s}=P_{0}+1645\frac{\sqrt{P_{0}\left(1-P_{o}\right)}}{n}$$

where *n* is the number of runs (number of panelists × number of replications). For each sample we used three replications. When an attribute overcomes the significance level, it is considered as significantly dominant for a given time point, rather than by chance. In this study, the significance level was calculated as *P*_*s*_ **= 32%. For data management during the sensory evaluation, we used FIZZ Sensory Analysis Software, Version 2.50b, Biosystèmes, France.

## 3 RESULTS

**Figure 1:**
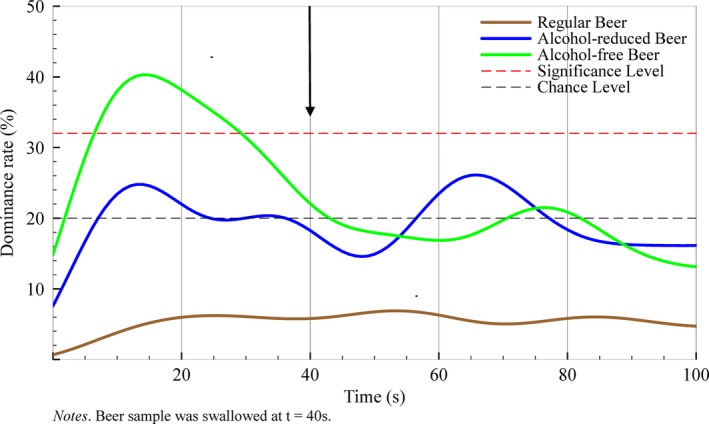
Flavor Life Cycle of worty flavor across three different beer types with varying alcohol content

For all regular beers, dominance rates for worty flavor were never higher than chance during consumption. In alcohol‐reduced beers, worty flavor never reached the level of significance while dominance rates were above chance before (6–24 s) and after swallowing (55–77 s). In alcohol‐free beers, worty flavor dominated the sensation prior to swallowing. Especially between 5 and 30 s, worty flavor was significantly dominant in alcohol‐free beers (*p* < .05). After swallowing, worty flavor did not show significant dominance rates (see Figure 1).

**Figure 2:**
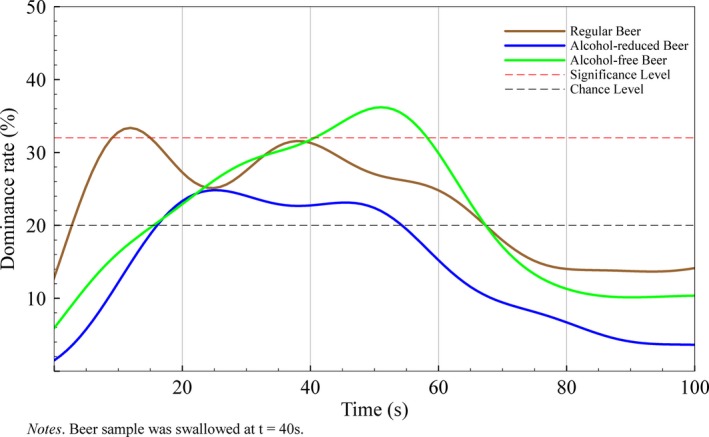
Flavor Life Cycle of malty flavor across three different beer types with varying alcohol content

In regular beers, dominance rates for malty flavor were significant between 6 and 14 s (*p* < .05) prior to swallowing, however the level of significance was never reached again until the end of consumption. In alcohol‐reduced beers, sensation for malty flavor was higher than the level of chance between 15 and 53 s, however, never reached the level of significance. In alcohol‐free beers, the level of significance for malty flavor was reached directly after swallowing (43 s) and remained until 57 s (*p* < .05). In the last third of the consumption (67–100 s), malty dominance rates dropped beyond the level of chance for all three beers (see Figure 2).

**Figure 3:**
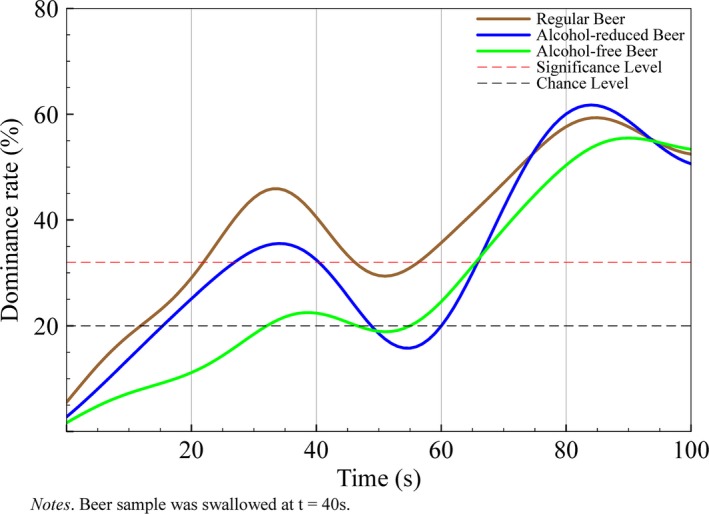
Flavor Life Cycle of bitterness flavor across three different beer types with varying alcohol content

Dominance rates for bitterness showed similar patterns across all beer products, increasing gradually prior to swallowing (<40 s), decreasing after swallowing (40–60 s), and in the last third of consumption level of significance is displayed for all beers (*p* < .05). In detail, bitterness dominance reached the level of significance between 23 and 45 s for regular beers and between 26 and 41 s for alcohol‐reduced beers. Between 46 and 55 s for regular beers and 42 and 64 s for alcohol‐reduced beers, dominance rates did not reach significance; however for both beer variations, bitterness was significantly dominant afterwards, until the end of consumption. For alcohol‐free beers, bitterness dominated significantly between 64 and 100 s (see Figure 3).

**Figure 4:**
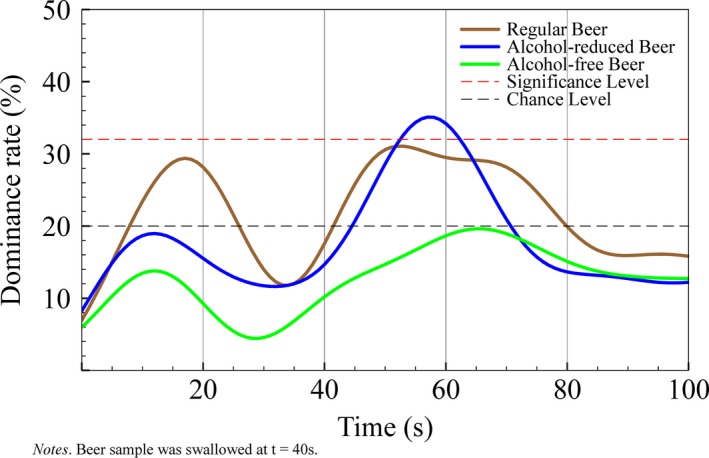
Flavor Life Cycle of astringency flavor across three different beer types with varying alcohol content

Astringency sensation was most pronounced in regular beers prior to swallowing and directly after swallowing; however, this did not reach significant levels during consumption. In alcohol‐reduced beers, astringency dominated significantly between 50 and 63 s (*p* < .05), however – from second 70 on – did not reach the level of chance. In alcohol‐free beers, astringency never reached the level of chance (see Figure 4).

**Figure 5:**
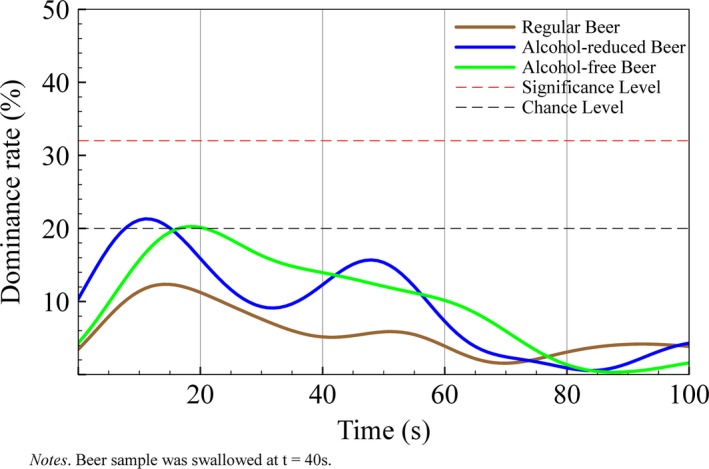
Flavor Life Cycle of fruity flavor across three different beer types with varying alcohol content

Fruity flavor was the least dominant flavor attribute in all beers. In regular beers, fruity flavor never reached the level of chance; in alcohol‐reduced beers and alcohol‐free beers, the level of chance is reached before swallowing, between 6 and 14 s for alcohol‐reduced beers and 16 and 21 s for alcohol‐free beers (see Figure 5).

## 4 DISCUSSION

Beer with varying alcohol content shows several differences in flavor dominance during the Flavor Life Cycle. For alcohol‐free beers, undesired by‐products such as worty‐off flavors have been reported as the result of dealcoholization methods (Sohrabvandi et al., 2010). Prior to swallowing, worty‐off flavor was most dominant in alcohol‐free beers, but not in alcohol‐reduced beers and in regular beers. This result is in line with previous research that have identified worty‐off flavor as dominant flavor for alcohol‐free beers (Blanco, Andres‐Iglesias, & Monero, 2016). Ethanol is relevant to form flavor characteristic in beer. In fact, worty off‐flavor dominance depends on the applied brewing processes that are either based on ethanol removal or reduction (Catarino & Mendes, 2011; Perpete & Collin, 1999). In our study, the undesirable worty off‐flavor was most pronounced in alcohol‐free beers, but only before swallowing. For consumers, this information might be highly valuable for their own drinking behavior. As such, to maximize pleasure and minimize the unpleasant experience, alcohol‐free beers should be swallowed faster to bypass worty‐off exposure allowing focusing on the flavor characteristics that arise after swallowing, such as malty flavor and bitterness.

Malty flavor is more dominant in regular beers prior to swallowing; however the increased dominance does only show between 6 and 14 s. After swallowing the beer sample, alcohol‐free beers showed some decreased dominance, but this leveled off around 20 s after swallowing. Malty flavor is caused by the presence of 3‐methylbutanal, 2‐methylbutanal and methional as the main contributors (Beal & Mottram, 1994). Compound amount depend on the amount and malt variety that is used for brewing. In our analysis, malty flavor dominated in regular and alcohol‐reduced beers prior to and in alcohol‐free beers after swallowing. This contradictory finding for malty flavor might therefore be a consequence of differences in malt processing during brewing rather than the alcohol content of the evaluated beers. Consumers who favor malty flavors but want to consume alcohol‐free beers might focus on post‐swallowing phase to maximize malty flavor experience during drinking.

Bitterness dominated the flavor profile after swallowing and showed the best congruency for all five different flavors among different types of beer. Bitterness dominance increased with time of consumption and alcohol content. Bitterness depends on the alcohol content present in beer as reported by Arrieta, Rodríguez‐Méndez, de Saja, Blanco, and Nimubona (2010), who showed that the percentage of alcohol in beer correlates well with the concentration of iso‐alpha‐acids, which contribute to approximately 80% of the bitter taste in beer. Associated with this is the somatosensory perception of astringency – that is characterized by drying and puckering of the mucosal surface within the mouth. Although not being perceived instantly, astringency evolves continually during consumption. François et al. (2006) identified the pH‐level as the most important factor for astringency to emerge. Low pH‐values result in higher astringency perception. In beer, astringency develops faster at pH 3.0 than at pH 5.0. High iso‐alpha acids concentration, which are associated with higher ethanol content result in increased sourness of the evaluated beer and therefore higher astringency dominance. As a consequence, astringency also depends on the alcohol content, even though only indirectly.

### 4.1 Implications for consumers, breweries and public health

Reducing the alcohol content without taking away the pleasure of consumption is one important strategy to move forward in the field of alcohol prevention in Public Health (Shield et al., 2016). Alcohol‐free beers have been available for several decades, however, alcohol‐reduced beers are considered as relatively new beverages that are consumed less commonly compared to regular beers. From a Public Health perspective, reducing the alcohol content for alcoholic beverages can accumulate lower total alcohol consumption over time and as a consequence drive positive health outcomes such as overall health, safety in the workplace or road traffic (Brányik et al., 2012).

Despite these positive aspects, research suggests that lower preference for beers with reduced alcohol is based on differences in product conceptualization with alcohol‐free beers eliciting rather functional consumption expectations compared to regular beers (Silva et al., 2016). Besides differences in ascribed expectations, differences in flavor (e.g., problematic off‐flavors) additionally shape consumer expectations of beer with lower alcohol. As shown in this study, worty‐off flavor is the most dominant flavor in alcohol‐free beers contributing to aberrant expectations for beer consumers. In contrast, bitterness shows good congruency in temporal dominance of the Flavor Life Cycle of beers with varying alcohol content during consumption.

From these initial descriptions of the main dominant flavor attributes, we propose that alcohol‐free beer consumers should consider focusing on the post‐swallowing phase during consumption. In detail, rather fast swallowing will decrease worty‐off experience and focusing on the post‐swallowing phase will increase malty flavor and favorable bitter flavor experience. With this brief behavioral advice, consumers are able to maximize their consumption experience of alcohol‐free beers. These brief advices potentially benefit breweries likewise. Marketing strategies that emphasize on “how to” consume different types of beers could draw on the findings of this study by using sensory marketing techniques. In addition, our findings are of importance to develop novel formulations and recipes that target the overall experience during the process of consumption. For this, preventing worty‐off flavor might still be the most challenging task in alcohol‐free beers. This holds true for alcohol‐reduced beer, although not as prominent as in alcohol‐free beers. To tackle this sensory problem, different technological approaches have been proposed (Perpète & Collin, 2000; Scanes, Hohmann, & Prior, 1998).

Besides technological modifications, breweries should consider emphasizing on congruencies rather than differences in their product lines in their marketing strategies, as marketed differences lead to differences in expectations, taste and potentially decreasing overall acceptance. Our study showed that bitterness dominance developed similarly over time of consumption, which might be a potential target for improving marketing strategies for the dedicated products.

### 4.2 Limitations and future research

In our study, we used the TDS method to assess flavor dominance during beer consumption. TDS is a merely descriptive and qualitative method. However, the strength of the TDS is, that it rules out the possibility of the so‐called “halo‐dumping effect” which is prevalent in other dynamic evaluation methods (e.g., Time Intensity Method) (Pineau et al., 2009). The novelty of our findings is limited to the applied method (TDS) and the beers used in our study (beers from Austrian breweries). In this investigation, only three beer brands were analyzed which limits our findings, even though we applied an aspired number of sessions as proposed by Pineau & Schlich (2015) and a sufficient list of attributes (Pineau et al., 2012). In addition, research using sensory methods should take a limited ecological validity into account when deducing potential implications for consumers. As recently pointed out by Jaeger et al. (2017), further studies in more natural situations are needed to increase the ecological validity of sensory studies in general. In this study, we presented a preliminary analysis on changes in flavor dominance over time, however future studies should provide data collected in more natural settings, likewise. Future studies should also analyze a higher number of brands for better comparability and might emphasize on other, nonflavor‐associated limitations for consumption of alcohol‐reduced beverages in general (e.g., social acceptance, self‐identity). Another avenue to investigate would be to analyze differences in temporal consumption pattern depending on time of the year or special occasions during the year (e.g., during catholic fasting).

### 4.3 CONCLUSION

Decreasing alcohol while increasing pleasure of beers with lower alcohol should be the ultimate goal for health conscious consumers, breweries and Public Health stakeholders. Beers with reduced alcohol display important differences during the Flavor Life Cycle that need to be addressed in future research. For now, consumers who want to enjoy beers with lower alcohol should consider changes in flavor perception during consumption to focus on the favored and defocus on the less favored flavors.

## AUTHOR CONTRIBUTIONS

DM, BM, and RS conceived of the study and RS carried it out. RS, MWR, and BM analyzed the data. BM, BW, and DM wrote the first draft of the manuscript, which JK and DM revised. All authors approved the manuscript as submitted.

## CONFLICT OF INTERESTS

The authors have no competing conflict of interest.
